# Exclusive liposuction with glandular tissue redistribution for severe gynecomastia: A case report

**DOI:** 10.1097/MD.0000000000041299

**Published:** 2025-01-17

**Authors:** Bingwen Yan, Xuezhe Hong, Dongyue Hao, Juan Zhang, Jiaomiao Pei, Liming Sun, Zhengqiang Cang, Yongjun Chen, Ying Ma, Baoqiang Song, Chaohua Liu

**Affiliations:** a Department of Plastic and Reconstructive Surgery, Xijing Hospital, Fourth Military Medical University, Xi’an, Shaanxi, China.

**Keywords:** gynecomastia, liposuction, minimally invasive surgery, Stab Flatten technique

## Abstract

**Rationale:**

Gynecomastia, characterized by abnormal enlargement of male breast tissue, can lead to significant psychological distress, particularly among younger men. Traditional surgical options, such as subcutaneous mastectomy and liposuction, often result in visible scarring and contour deformities. This study introduces the “Stab Flatten” technique, a novel, minimally invasive approach for treating severe gynecomastia, designed to preserve chest aesthetics while minimizing postoperative complications, including scarring and contour irregularities.

**Patient concerns:**

A 28-year-old male with a 15-year history of progressive bilateral breast enlargement presented with psychological distress and concerns about the cosmetic outcomes of traditional surgery. He sought a minimally invasive procedure to address both physical discomfort and aesthetic concerns, aiming for minimal scarring.

**Diagnoses:**

The patient was diagnosed with grade III gynecomastia according to Simon classification, characterized by significant glandular hypertrophy and excess skin. Diagnostic imaging and laboratory tests confirmed the absence of underlying medical conditions.

**Interventions:**

The patient underwent the “Stab Flatten” technique, which involved exclusive liposuction with glandular tissue redistribution. A 4-mm incision was made at the inframammary crease, and power-assisted liposuction was used to flatten fibroglandular tissue without excision. Postoperative care included a compression garment for 6 months.

**Outcomes:**

At the 6-month follow-up, the patient showed excellent cosmetic results, with significant improvement in breast contour and symmetry. There were no complications such as hematoma, seroma, or nipple-areola complex deformities. The patient reported high satisfaction with the aesthetic outcome, alongside improved psychological well-being and physical comfort.

**Limitations:**

This study is limited by its single-patient case report, which restricts the generalizability of the findings. Long-term outcomes of the “Stab Flatten” technique require further validation through larger, prospective studies with more diverse patient populations. Additionally, the technique may not be applicable to all grades of gynecomastia or patients with more complex conditions.

**Lessons:**

The “Stab Flatten” technique offers an effective, minimally invasive alternative for treating severe gynecomastia, providing excellent cosmetic and functional outcomes while minimizing scarring. This method may enhance patient satisfaction and reduce recovery times compared to traditional surgical approaches. However, further studies with larger cohorts are necessary to validate its efficacy and generalizability.

## 1. Introduction

Gynecomastia is characterized by an abnormal increase in fibroglandular tissue in the male breast, typically exceeding 2 cm and palpable beneath the nipple and areola.^[1]^ While traditionally more common in adolescents and older adults, recent trends indicate a significant rise in its overall prevalence, now affecting up to 70% of the male population.^[2–4]^ This condition is increasingly prompting men to seek corrective surgery.^[5,6]^ The psychological impact of gynecomastia is profound, particularly among younger males, where it often leads to emotional distress, social discomfort, and reduced quality of life.

Surgical intervention for gynecomastia is typically pursued for both symptomatic relief and cosmetic improvement, particularly when medical management fails to yield satisfactory results.^[7]^ Minimally invasive techniques offer the advantage of quicker recovery, reduced pain, and minimal scarring. Procedures such as subcutaneous mastectomy, ultrasound-assisted liposuction, and suction-assisted lipectomy have been utilized in clinical practice. Patients with more pronounced cases of gynecomastia often face challenges such as contour irregularities, periareolar wrinkling, and noticeable scarring. Consequently, many plastic surgeons are developing innovative techniques designed to effectively address severe gynecomastia while minimizing visible scars.

## 2. Case report

**Figure 1.**
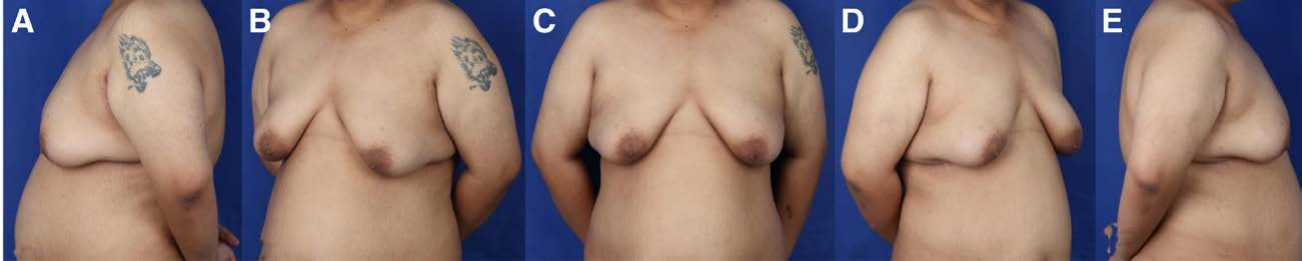
Preoperative photograph of a 28-year-old patient with grade III gynecomastia, demonstrating severe breast enlargement with pronounced glandular tissue hypertrophy and significant ptosis. The patient’s chest contour is notably distorted, with excessive subcutaneous adipose deposition and stretched overlying skin. This presentation exemplifies the advanced stage of gynecomastia, which often requires surgical intervention due to the significant physical and psychological burden imposed by the condition. Preoperative images captured from 5 different angles (A–E).

A 28-year-old male patient presented with a 15-year history of progressive bilateral breast enlargement, leading to significant aesthetic concerns and psychological distress. The patient explicitly denied the use of anabolic steroids, hormone therapies, or a history of rapid weight fluctuations. Additionally, there was no family history of breast or endocrine disorders. The patient’s overall health status was unremarkable, with no notable comorbidities or prior surgical interventions. His lifestyle habits were aligned with health recommendations, as he abstained from smoking, alcohol consumption, and recreational drug use. On clinical examination, pronounced bilateral breast hypertrophy was observed. The patient’s body mass index was measured at 34.6, which is consistent with a classification of obesity (Fig. 1).

The diagnosis of grade III gynecomastia was confirmed based on Simon classification, characterized by marked breast enlargement, glandular hypertrophy, and significant skin redundancy. Ultrasound evaluation revealed dense glandular tissue with no evidence of malignancy. Comprehensive laboratory assessments, including liver function tests, hormonal profiles (testosterone, estradiol, luteinizing hormone, follicle-stimulating hormone, and prolactin), and renal function, were all within normal physiological ranges, effectively ruling out secondary etiologies for the gynecomastia. The patient expressed a strong preference for a minimally invasive approach, prioritizing reduced scarring and optimal aesthetic outcomes. He emphasized his desire for a surgical intervention that would not only address the physical discomfort and psychological burden caused by the gynecomastia but also preserve chest contour aesthetics with minimal visible postoperative scars.

In alignment with the patient’s specific concerns, we introduced an innovative approach by performing exclusive liposuction, without excision of glandular tissue or skin. This was combined with the concept and technique of glandular tissue redistribution. Following a 6-month postoperative evaluation, the results demonstrated excellent clinical outcomes.

We refer to this novel surgical technique as the Stab Flatten method. Prior to surgery, the patient is positioned upright to accurately mark the areas for liposuction, extending beyond the nipple-areola complex to include regions of adipose accumulation. This ensures comprehensive contouring and smooth integration of the nipple-areola complex with adjacent tissues, preventing a recessed appearance postoperatively.

Following anesthesia induction, the patient is repositioned supine, and a 4-mm stab incision is made at the inframammary crease along the anterior axillary line. A tumescent solution (1000 mL 0.9% sodium chloride, 20 mL 2% lidocaine, 2 mL 1:1000 epinephrine per breast) is infiltrated into the subcutaneous and deeper breast tissues.

Power-assisted liposuction (PAL) is employed to address excess breast volume, particularly targeting subcutaneous fat and subdermal layers beneath the nipple-areola complex. This method facilitates significant volume reduction while enhancing postoperative aesthetic outcomes.

The Stab Flatten technique utilizes narrow-gauge liposuction cannulas to precisely disrupt the subdermal attachments of fibrous breast tissue, as well as the lactiferous duct connections to the nipple. Rather than removing the tissue, this approach fragments it via the liposuction incision. Special care is taken to address the subdermal tissues beneath the nipple-areola complex, preventing the development of concave deformities. In cases where fibroglandular breast tissue is present, it is carefully flattened to improve aesthetic outcomes.

A liposuction needle is inserted through the incision, and the fibroglandular tissue is methodically fragmented using a “stab” motion, while the skin is simultaneously pinched to enhance precision. This process is continued until the ribs are palpable and no glandular texture remains on compression, at which point the procedure is concluded. Notably, no tissue is excised during the procedure; the tissue is merely fragmented and flattened by the liposuction cannula.

Extreme care is taken to prevent depressions under the nipple-areola complex, and symmetry is assessed postliposuction. The incisions are closed with an inverted deep dermal 5-0 Monocryl suture, reinforced with #7-0 nylon sutures. Drains are not required. Postoperative care involves the use of a compression garment for 6 months to facilitate optimal skin adherence and recovery. Patients can typically resume normal activities within 3 weeks.

At the 6-month follow-up, the patient demonstrated significant improvements in both aesthetic and functional aspects. The breast contour and symmetry were markedly enhanced, with a natural and smooth chest profile achieved. There were no complications such as hematoma, seroma, or skin necrosis.

Importantly, there were no concave deformities or visible scarring, especially around the nipple-areola complex, which is a common concern with traditional gynecomastia surgeries. The patient reported high satisfaction with the cosmetic results, highlighting the improved overall chest appearance and the absence of visible scars. Psychological well-being also showed improvement, with the patient expressing a reduction in body image-related distress.

Additionally, the patient did not require any drain placement postoperatively, and no major postoperative issues were observed. The compression garment was effective in supporting optimal skin adherence, and the patient resumed normal activities within 3 weeks.

**Figure 2.**
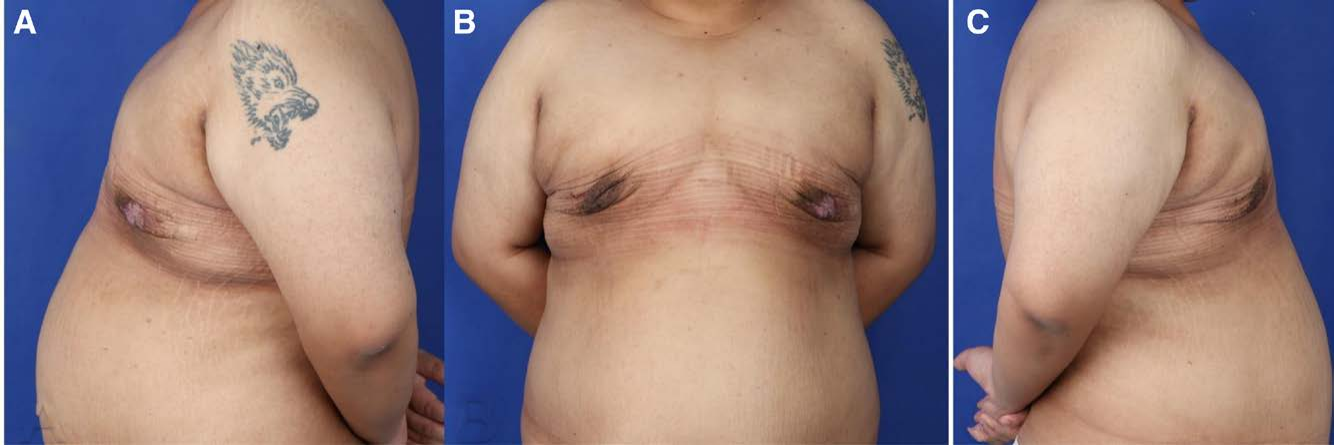
Postoperative images at 3 months following surgical correction of grade III gynecomastia using the “Stab Flatten” technique, captured from 3 different angles (A–C). The images show significant improvement in chest contour, with effective reduction of both glandular tissue and adipose volume. Skin retraction is well-aligned, and the natural masculine chest appearance has been restored.

**Figure 3.**
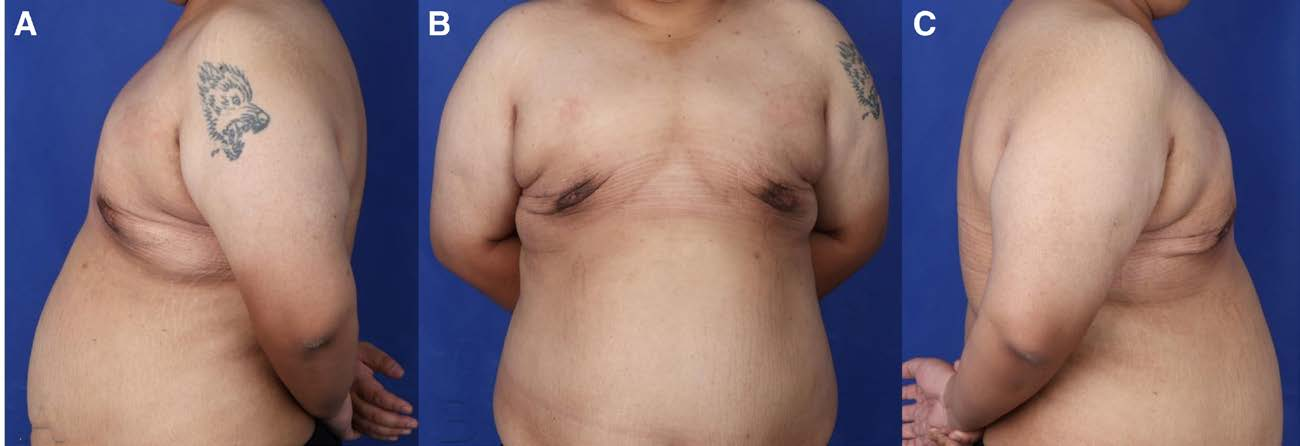
Postoperative photographs taken 6 months after surgery using the “Stab Flatten” technique, viewed from 3 different angles (A–C), showing significant improvement in chest contour and overall appearance. The procedure effectively reduced breast volume, achieving a smooth and natural result. The skin has retracted well, with minimal scarring, which is virtually imperceptible, reflecting the success of the “Stab Flatten” method in delivering both functional and aesthetic benefits.

In terms of long-term outcomes, the patient showed sustained improvement in breast contour with no significant changes or complications at the 6-month mark. The results suggest that the “Stab Flatten” technique is an effective, minimally invasive solution for severe gynecomastia, providing excellent cosmetic and functional outcomes (Figs. 2 and 3).

## 3. Discussion

Gynecomastia arises from an imbalance between estrogen and androgen levels or heightened breast tissue sensitivity to estrogen, leading to abnormal mammary gland and/or adipose tissue growth.^[8]^ Various grading systems have been introduced to evaluate the severity of gynecomastia, including those by Simon,^[9]^ Rohrich,^[10,11]^ Waltho,^[2]^ and Webster.^[12]^ Among these, Simon classification is most widely used and divides gynecomastia into 4 stages: grade I, mild hypertrophy without excess skin; grade IIa, moderate hypertrophy without excess skin; grade IIb, moderate hypertrophy with minor skin redundancy; and grade III, severe hypertrophy with significant skin redundancy. Gynecomastia can greatly affect quality of life, contributing to psychological distress, such as depression and anxiety, and negatively influencing daily functioning. Therefore, timely and effective intervention is essential to restore normal chest contour and improve patient outcomes.

Surgical intervention is widely regarded as the most effective approach for alleviating the psychological burden associated with gynecomastia. Various surgical techniques have been developed for male breast reduction, including liposuction, excision, a combination of liposuction and excision, as well as minimally invasive methods. Liposuction can be executed using multiple techniques, such as negative pressure-assisted, ultrasound-assisted, radiofrequency-assisted, or laser-assisted methods.^[13]^ Direct excision is typically performed through incisions in the areolar or periareolar regions.^[14]^ Furthermore, minimally invasive approaches like breast endoscopy and vacuum-assisted gyrotomy have also been utilized.^[15,16]^

Despite their widespread adoption, these conventional approaches exhibit inherent limitations and may fall short of fully addressing the unique needs and expectations of our patients. In light of this, we propose an innovative surgical method specifically designed to address key aesthetic and functional challenges, such as breast ptosis and minimizing postoperative scarring. This technique emphasizes a patient-centered approach, focusing on achieving optimal results with fewer complications. By employing individualized, precision-tailored procedures, we ensure the best possible outcomes for this patient.

In our study, liposuction served as a cornerstone in the surgical management of gynecomastia, with a particular focus on customizing the procedure to accommodate the distribution and specific characteristics of the patient’s adipose tissue. Notably, we preserved retroglandular fat to prevent the flap from adhering directly to the deep fascia, which could otherwise result in contour deformities during muscular contractions. This approach is particularly important in overweight patients, where maintaining a thicker flap ensures a smoother and more natural contour that harmonizes with their chest profile.

One of the most challenging aspects of gynecomastia surgery is distinguishing between adipose and glandular tissues preoperatively. To address this, we advocate initiating the procedure with liposuction, as it facilitates a clearer definition of peripheral contours and glandular tissue boundaries. PAL, widely available and highly effective, plays a pivotal role in these cases by streamlining fat removal and enhancing the overall outcome.^[17]^

A critical balance must be struck between completely excising glandular tissue to prevent recurrence and avoiding complications such as nipple retraction, especially in patients with larger, fatty breasts. This is where the Stab Flatten technique becomes invaluable, as it effectively addresses potential depressions, asymmetries, or irregularities in the nipple-areola complex. In our case, the combination of liposuction and the Stab Flatten technique was chosen for multiple reasons. First, PAL reduces the physical strain typically associated with adipose tissue removal through dense breast parenchyma, thereby increasing procedural efficiency. Additionally, the use of small-diameter liposuction cannulas allows for effective contouring of fibroglandular tissue beneath the nipple-areola complex through a minimal, peripherally placed incision, a level of precision not achievable with conventional methods using clamps or scalpels.

Compared with traditional open surgical approaches, this technique offers several significant advantages. These include minimal incisions, reduced trauma to surrounding tissues, enhanced viability of the nipple-areola complex, and expedited recovery times. One particularly notable benefit is the absence of noticeable scarring; none of our patients reported dissatisfaction with scarring, as there were no visible circumareolar scars and only minimal scarring at the incision site. From an economic perspective, the technique is cost-effective, relying solely on basic suction technology without the need for more complex modalities like ultrasound or vibration amplification.

Moreover, the avoidance of drains postoperatively, in line with current evidence suggesting limited benefits from their use in the reduction of mammoplasty, contributed to shorter hospital stays and lower overall costs.^[18]^ The absence of drainage further simplified the postoperative course, with no increase in complications such as hematoma or seroma. Given the palpable nature of fibroglandular breast tissue, we found that minimal access excision techniques under direct vision or with endoscopic assistance were unnecessary. Instead, our approach relied on tactile feedback, which proved sufficient for precise excision without bleeding complications, thanks to the inclusion of epinephrine in the tumescent solution.

Importantly, none of our patients experienced concave deformities in the nipple-areola complex, a common complication associated with open excision techniques. This favorable outcome can be attributed to careful tissue preservation and tension distribution, which prevented excessive removal of tissue beneath the nipple-areola complex.

For patients with severe, grade III gynecomastia, mastectomy with skin resection is often the treatment of choice.^[19]^ However, our approach offers a less invasive alternative with promising results, although further studies involving larger cohorts are necessary to confirm these findings.

It is worth noting that this technique requires advanced surgical skills and is not recommended for novice surgeons due to the complexity of glandular tissue manipulation. In some cases, optimal results may necessitate combining liposuction with other techniques such as skin-sparing mastectomy or pedicled nipple-areola complex flaps.

## 4. Limitations

This case report has several limitations. First, it is based on a single patient, limiting the generalizability of the results. Larger studies are needed to confirm the findings. Second, the follow-up period was only 6 months, so long-term outcomes and the sustainability of results remain uncertain. Third, the “Stab Flatten” technique was applied to grade III gynecomastia, and its efficacy in milder cases or those with significant skin excess is unclear. Additionally, a comparative analysis with other surgical methods was not performed, which would be valuable for a more comprehensive evaluation. Finally, the technique requires advanced surgical skills, which may limit its use by less experienced surgeons. Further studies are needed to address these issues.

## 5. Conclusion

The present case report highlights the significant therapeutic value of the “Stab Flatten” technique for the treatment of severe gynecomastia. This innovative approach, involving glandular tissue redistribution through selective liposuction and a minimally invasive stab incision, demonstrates both efficacy and safety. By preserving the natural contour of the chest and minimizing visible scarring, this method offers a novel solution for patients with severe presentations, addressing both functional and aesthetic concerns while reducing the risk of complications associated with traditional surgical approaches.

## Author contributions

**Writing – original draft:** Bingwen Yan, Xuezhe Hong.

**Writing – review & editing:** Bingwen Yan.

**Data curation:** Dongyue Hao, Juan Zhang, Jiaomiao Pei, Liming Sun, Zhengqiang Cang, Yongjun Chen.

**Investigation:** Dongyue Hao, Juan Zhang, Jiaomiao Pei, Liming Sun, Zhengqiang Cang, Yongjun Chen.

**Conceptualization:** Ying Ma, Baoqiang Song, Chaohua Liu.
